# Prophylactic effects of Wormwood on lipid peroxidation in an animal model of lead intoxication

**DOI:** 10.4103/0971-4065.42333

**Published:** 2008-04

**Authors:** O. Kharoubi, M. Slimani, A. Aoues, L. Seddik

**Affiliations:** Department of Biology, Faculty of Science; Laboratory of Biochemistry. University of Es-senia, Oran, Algeria

**Keywords:** *Artemisia absinthium* extract, atpase, carbonyl, kidney, lead acetate, lipids, liver, TBARS

## Abstract

The ability of Wormwood (*Artemisia absinthium* L.) extract (A.Ab) to restore membrane-bound enzymes like Na^+^-K^+^-ATPase, Ca^++^-ATPase, Mg^++^-ATPase, and oxidative damage induced by lead were investigated. Rats were exposed to lead acetate (750 ppm) for 11-weeks and treated during 4-weeks with A.Ab. Lipid levels, ATPase activity, thiobarbituric acid reactive substances (TBARS), and proteins carbonyl were estimated. In liver and kidney, lead acetate inhibited membrane-bound enzymes and increased (*P* < 0.05) the levels of cholesterol, triglycerides, free fatty acids, phospholipids, TBARS, and carbonyl proteins. After 4 weeks, the intoxicated group who received A.Ab showed a significant reduction in TBARS and carbonyl levels in liver and kidney compared to group exposed to lead. A.Ab restored the levels of membrane-bound enzymes and lipid levels to near normal. These results indicate that aqueous Wormwood extract had a significant antioxidant activity and protect liver and kidney from the lead-induced toxicity.

## Introduction

Lead (Pb) is a ubiquitous environmental toxin. Exposure to lead has been shown to disrupt many processes in the liver and kidney.^1^ Several molecular mechanisms that result in damage to cellular membrane lipids leading to membrane fragility and permeability are thought to exist. One possibility is the disruption of the prooxidant/antioxidant balance,^2^ which can lead to liver and kidney injury. Lead is also reported to release free radicals (hydroxyl)^3^ thereby stimulating the process of lipid peroxidation.^1^ The assumption of oxidative stress as a mechanism in lead toxicity suggests that antioxidants might play an important role in therapy. Oxidative stress is defined as the imbalance between reactive oxygen species (ROS) production and natural antioxidants in biological systems, which leads to the damage of macromolecules such as lipids, proteins, carbohydrates, RNA, and DNA. Lead is reported to have an inhibitory action on the membrane bound enzymes such as Na^+^-K^+^-ATPase, Ca^++^-ATPase, and Mg^++^-ATPase in various vital organs.^4^ The effect of *Artemisia absinthium* L. (Wormwood) extract on the lead-induced changes on lipids composition, antioxidant status, and membrane-bound enzymes, which is a consequence of oxidative stress have not been studied. The usefulness of antioxidants alone or along with chelation therapy has yet not been extensively investigated. It was shown that Wormwood (*Artemisia absinthium* L.) extract have high contents of total phenolic compounds and total flavonoids, suggesting that these compounds contribute to the antioxidative activity^5^; phenolic substances such as flavonols, cinnamic acids, coumarins, and caffeic acids or chlorogenic acids are believed to have antioxidant properties, which may play an important role in protecting cells and any organ from oxidative degeneration.^6^,^7^ In addition polyphenols, which constitute the active substances found in many medicinal plants, play a role in the prevention of various diseases associated with oxidative stress^8^–^10^ and modulate the activity of a wide range of enzymes and cell receptors.^11^ Wormwood extract has been recommended for gastric pain, as a cardiac stimulant, and for restoration of declining metal functions. *Artemisia absinthium* L. extract has been reported to exhibit anti-inflammatory and antipyretic effect, since this plant contain azulenes.^12^ In addition, protective effects of this extract have been experimentally demonstrated on hepatic damage.^13^,^14^ Thus, the aim of this investigation is to evaluate the effect of the aqueous extract of *Artemisia absinthium* L. on lead-induced dysfunction of liver and kidney of rats exposed to lead, to restore lipids and proteins peroxidation accumulation, the tissue membrane bound enzymes like Na^+^-K^+^-ATPase, Ca^++^-ATPase and Mg^++^-ATPase, and various lipids in vital organs.

## Materials and Methods

### Preparation of wormwood (*Artemisia absinthium* L.) plant extracts (A.Ab)

Whole plants of *Artemisia absinthium* L. were collected from Mecheria, Algeria in the month of May. The plant was identified and authenticated at the Herbarium of Botany Directorate in Es-Senia (Oran) University. Five hundred grams of whole Wormwood plants were extracted with 1.5 l of distilled water by the method of continuous hot extraction at 60°C twice for 30 min, and the filtrate was lyophilized. The residue collected (yield 75 g) were stored at −20°C; when needed the extract was dissolved in distilled water and used in the investigation.

In the experiment, a total of 30 male Wistars rats (18 intoxicated rats, 12 normal rats) were used. The rats were housed five per cage and had free access to food and water, except during testing. They were exposed to a 14-10 h light-dark cycle and the room temperature was controlled at 23 ± 2°C. Animals were first exposed to Pb at the age of 2 weeks, when they weighed 40 ± 6 g. Experiments were performed during 11 weeks:

- *Untreated group*: rats that received water during 11-weeks.
- *Pb group*: Rats exposed to Pb (750 ppm, in the form of Pb acetate in their drinking water *ad libitum*) for 11-weeks.
- *Untreated/A.Ab group*: rats exposed to Pb that later receiving aqueous Artemisia absinthium *L.* extracts (A.Ab) (200 mg/kg, in their drinking water *ad libitum*) for 4-weeks.
- *Pb/water group*: Rats exposed to Pb for 11-weeks, receiving water for four additional weeks.
- *Pb/A.Ab groups*: rats exposed Pb who later received aqueous *Artemisia absinthium* L. extract (A.Ab) (200 mg/kg, in their drinking water *ad libitum*) for 4-weeks.

Animals were sacrificed by cervical decapitation under pentobarbital sodium anesthesia (60 mg/kg). Tissue samples of kidneys and liver were collected from all the animals, rinsed in ice cold saline (0.9% sodium chloride), blotted on a filter paper and weighed.

The body weight (BW) of each animal was noted before treatment. The weight of liver and kidney of respective groups of animals was recorded. From these values the organo-somatic index (OSI) was calculated by the following formula:

$$\mathrm{Organo}-\mathrm{somatic index}=\frac{\mathrm{Weight (g) of the organ}}{\mathrm{Total body weight (g)}}\mathrm{\times 100}$$

#### Postmitochondrial supernatant preparation (PMS)

Kidneys and liver were homogenized in ice-cold isolation medium containing 0.32 M sucrose, 0.5 mM EDTA, 10 mM Tris-HCl (pH 7.2) using a Teflon/glass homogenizer. The homogenates were centrifuged at 800 × *g* for 5 min at 4°C to separate the nuclear debris. The supernatant so obtained was centrifuged at 10.000 × *g* for 25 min at 4°C to get the postmitochondrial supernatant which was used to assay enzymes activity.

Before sacrifice, rats were kept individually in metabolic cages for 72 h to collect urine for estimation of renal function. Serum samples were assayed for blood urea nitrogen (BUN) and serum creatinine by using standard diagnostic kits.

Serum alanine aminotransferase (ALT) and serum aspartate aminotransferase (AST) were estimated by International Federation of Clinical Chemistry.^15^ Serum bilirubin was estimated by Diazo method.^16^

The malondialdehyde (MDA) content, a measure of lipid peroxidation, was assayed in the form of thiobarbituric acid reacting substances (TBARS) by method of Ohkawa *et al.*^17^ Briefly, the reaction mixture consisted of 0.2 ml of 8.1% sodium lauryl sulfate, 1.5 ml of 20% acetic acid solution adjusted to pH 3.5 with sodium hydroxide and 1.5 ml of 0.8% aqueous solution of thiobarbituric acid was added to 0.2 ml of 10%(w/v) of PMS. The mixture was brought up to 4.0 ml with distilled water and heated at 95°C for 60 min. After cooling with tap water, 1.0 ml distilled water and 5.0 ml of the mixture of *n*-butanol and pyridine (15:1 v/v) were added and centrifuged. The organic layer was taken out and its absorbance was measured at 532 nm. TBARS were quantified using an extinction coefficient of 1.56 × 105 M^−1^/cm^−1^ and expressed as nmol of TBARS per mg protein. The protein was estimated using Lowry *et al.* method.^18^

Liver and kidney carbonyl formation was measured using 2,4-dinitrophenylhydrazine (DNPH) as a reagent according to Levine *et al.*,^19^ with some modifications. Briefly, 500 mg of frozen tissue samples were homogenized at 0-4°C in 5 mmol/l potassium phosphate buffer (pH 7.4; weight: volume = 1:10) including 0.1% Triton X and the protease inhibitors leupeptin (1.0 mg/l), pepstatin (1.4 mg/l), and aprotinin (1.0 mg/l). The homogenate was centrifuged at 500 × *g* for 3 min and the 900-µl supernatant was transferred to a microcentrifuge tube including 100 µl of 10% streptomycin sulfate (in 50 mmol/L HEPES). The samples were vortexed vigorously and incubated at room temperature for 15 min before centrifugation at 6000 × *g* for 10 min at 4°C and the supernatant was used. After reacting with 10 mmol/l DNPH: 2 mol/l HCl in the dark, protein was precipitated with 20% trichloric acid followed by centrifugation at 10.000 × *g* for 15 min. The pellets were washed thrice to remove excess DNPH, suspended in 6 mol/l guanidine HCl (in 20 mmol/l KH_2_PO_4_, pH 2.3), vortexed and allowed to dissolve overnight. The absorbance of the samples was measured at 366 nm. Carbonyl content was calculated using a molar absorption coefficient of 22.0 (mmol/L)^−1^cm^−1^.

Lipids were extracted from tissues by the method of Folch *et al.*,^20^ using chloroform methanol mixture (CHCl_3_:MeOH) (2:1 v/v). The total cholesterol was estimated by the method of Zlatkis *et al.*^21^ To 0.1 ml of the lipid extract, 9.9 ml of ferric chloride-acetic acid reagent was added and allowed to stand for 15 min and then centrifuged. To 5 ml of the supernatant, add 3 ml of H_2_SO_4_. The colour developed was read after 20 min at 560 nm against a reagent blank. Values were expressed as mg/100 g tissue. Triglycerides were estimated by the method of Foster and Dunn.^22^ To an aliquot of lipid extract, evaporated to dryness. 0.1 ml of methanol was added followed by 4 ml of isopropanol. 0.4 g of alumina was added to all the tubes and shaken well for 15 min. Centrifuged and then 2 ml of the supernatant was transferred to labeled tubes. The tubes were placed in a water bath at 65°C for 15 min for saponification after adding 0.6 ml of the saponification reagent followed by 0.5 ml of acetyl acetone reagent. After mixing, the tubes were kept in a water bath at 65°C for 1 h, the contents were cooled and absorbance was read at 420 nm. The triglyceride content was expressed as mg/100 g tissue. Phospholipids content was determined by the method of Zilversmit *et al.*^23^ To extract 0.1 ml of lipid, 1 ml of 5 N H_2_SO_4_ and 1 ml of concentrated nitric acid were added and digested to a colorless solution. The phosphorus content in the extract was determined by the method of Fiske and Subba Row.^24^ The values were expressed as g/100 g tissue. Free fatty acids were estimated by the method of Falholt *et al.*^25^ 0.1 ml of lipid extract was evaporated to dryness. 1 ml of phosphate buffer, 6 ml of extraction solvent, and 2.5 ml of copper reagent were added. All the tubes were shaken vigorously. Two hundred milligrams of activated silicic acid was added and left aside for 30 min. The tubes were centrifuged and 3 ml of the copper layer was transferred to another tube containing 0.5 ml of diphenyl carbazide and mixed carefully. The absorbance was read at 550 nm immediately. The amount of free fatty acids was expressed as mg/100 g tissue.

The Na^+^-K^+^ and Mg^++^ ATPase activities were assayed according to Kaplay^26^ and the inorganic phosphate was estimated by the method of Taussky and Shorr.^27^ The difference in the activity of the enzyme in the absence and presence of ouabain was taken as Na^+^-K^+^ ATPase and Mg^++^ ATPase activity, respectively. The enzyme activity was expressed as nanomoles of Pi liberated/min/mg protein. Ca^++^ ATPase activity was assayed by the method of Samaha and Yunis.^28^ The homogenates of liver and kidney were prepared in a histidine buffer (pH 7.2) containing 0.32 M sucrose and 0.01 M histidine, centrifuged for 30 min, and the resultant supernatant was used for enzyme assay. The amount of inorganic phosphate liberated was measured by the method of Taussky and Shorr.^27^ The enzyme activity was expressed as nanomoles of Pi liberated/min/mg protein.

Pb concentration was determined in blood and urine by atomic absorption spectrophotometry with a Zeeman-corrected graphite furnace (Model Spectra AA-220Z) and values were expressed in µg/dl in blood and µg/day in urine.

*Statistical analysis:* The mean ± SEM values were calculated for each group to determine the significance of inter group difference. Each parameter was analyzed separately using one way analysis of variance (ANOVA). To find the difference between the groups Student's ‘*t*’-test was used. *P* values < 0.05 were considered to be significant.

## Results

A significant difference in blood and urinary lead concentration was observed between Pb group (PbU = 6.94 ± 1.7 µg/day, PbB = 55.62 ± 6.30 µg/dl) at 11-weeks of intoxication compared to Pb/water (PbU = 2.11 ± 1.23 µg/day, PbB = 22.3 ± 5.78 µg/dl) and Pb/A.Ab groups (PbU = 1.12 ± 1.48 µg/day, PbB = 15.29 ± 6.21 µg/dl). Statistical significant differences in the BW were observed between Pb and untreated groups during 11-weeks of intoxication. In Pb group BW values were diminished by 28% compared with untreated group (*P* < 0.05) [Table 1]. In liver and kidney OSI were increased significantly in Pb compared to untreated group. After 4 weeks treatment with A.Ab extract, the OSI of liver was lowered significantly in intoxicated groups receiving A.Ab extract (Pb/A.Ab group) compared to untreated group, whereas in kidney, no difference was noted between Pb/A.Ab and untreated group [Table 1]. Pb caused a marked rise in serum levels of ALT (untreated = 42 IU/L) and AST (untreated = 128 IU/L) demonstrating a marked liver damage. Treatment with A.Ab extract decreased the elevated levels of ALT and AST in serum (*P* < 0.05) [Table 2]. A.Ab extract also attenuated the Pb-induced elevated levels of total bilirubin (control = 0.176 mg/dl). Significant elevation in BUN (untreated = 4.21 mg N/ml) and creatinine (untreated = 22.3 µmol/ml) in serum indicates kidney damage [Table 2].

**Table 1:** Body weight and organo-somatic index

		At 11 weeks		At 4 weeks		
			
	Organ	Untreated	Pb	Untreated/A.Ab	Pb/water	Pb/A.Ab
Body weight (g)		323.5 ± 22.1	254.2 ± 17.9	375.8 ± 27.2	327.3 ± 14.9	305.7 ± 28.8
Organo-somatic index	Liver	2.73 ± 0.11	0.50 ± 0.04	2.99 ± 0.19*	0.68 ± 0.07*	2.22 ± 0.09*,†
	Kidney	0.37 ± 0.07*,†	2.55 ± 0.21†	0.56 ± 0.10	2.37 ± 0.13*,†	0.48 ± 0.11†

Values are expressed as mean ± SEM (*n* = 6).

* *P* < 0.05, Pb group, untreated/A.Ab group, Pb/water group, and Pb/A.Ab group vs. untreated group (Control),

† *P* < 0.05, untreated/A.Ab group, Pb/water group, and Pb/A.Ab group are compared vs. Pb group. (Student's ‘*t*’-test)

**Table 2:** Effect of Wormwood extract on lead acetate induced rise in AST, ALT, total bilirubin, and BUN and creatinine

	At 11 weeks		At 4 weeks		
		
	Untreated	Pb	Untreated/A.Ab	Pb/water	Pb/A.Ab
ALT (IU/L)	100	174.15 ± 21.47*	108.91 ± 11.22†	162.45 ± 15.61*,c	138.24 ± 17.02*,†
AST (IU/L)	100	199.04 ± 18.56*	124.26 ± 19.46†	178.63 ± 25.21*,†	144.32 ± 14.76*,†
Bilirubin total (mg/dl)	100	175.21 ± 11.66*	110.15 ± 10.66†	166.63 ± 15.29*	139.45 ± 11.45*,†
BUN (mg N/ml)	100	175.05 ± 11.23	107.61 ± 9.23	161.28 ± 22.86	135.55 ± 12.88
Creatinine (µmol/ml)	100	197.32 ± 15.54	102.22 ± 12.32	169.55 ± 37.63	145.95 ± 29.68

Values are expressed as percent response compared to untreated rats (*n* = 6).

* *P* < 0.05, Pb group, untreated/A.Ab group, Pb/water group, and Pb/A.Ab group vs. untreated group (control),

† *P* < 0.05, untreated/A.Ab group, Pb/water group and Pb/A.Ab group are compared vs. Pb group (Student's ‘*t*’-test)

**Fig. 1:**
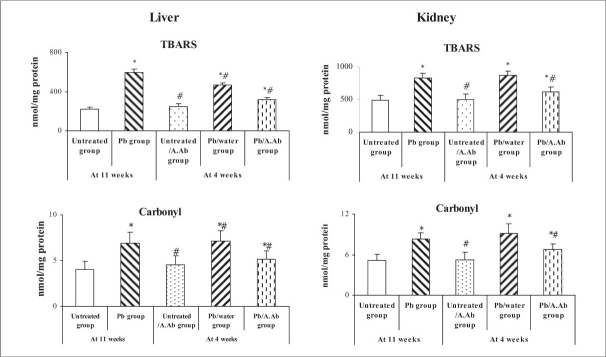
TBARS and carbonyl level in liver and kidney - Effect of wormwood extract on TBARS and carbonyls levels in Liver and Kidney. Values expressed as mean ± SEM (n=6). *P<0.05, Pb group, Untreated/A.Ab group, Pb/water group and Pb/A.Ab group were compared vs. Untreated group (as Control). # P<0.05, Untreated/A.Ab group, Pb/water group and Pb/A.Ab group are compared vs. Pb group (Student's ‘*t*’ test).

Lead exposure caused a marked lipid peroxidation and protein carbonyl accumulation in both liver and kidney. 4-weeks oral feeding of A.Ab per se (200 mg/kg) had a significant effect on alteration of both hepatic or renal TBARS and carbonyl levels. The A.Ab decreased the level of lipid peroxidation and carbonyl values in liver (-49%, -35% and -25%, -28%, respectively) and kidney (by -31%, -34% and -18%, -26%, respectively) compared to Pb and Pb/water groups (*P* < 0.05) [Fig. 1].

The membrane bound Na^+^-K^+^-ATPase, Ca^++^-ATPase, and Mg^++^-ATPase levels in liver and kidney decreased significantly (*P* < 0.05) in lead exposed (Pb group) animals as compared to untreated group [Table 2]. Furthermore, in Pb compared to untreated group, there was a significant increase in levels of cholesterol, triglyceride, free fatty acids and phospholipids in liver and kidney [Tables 3 and 4]. Treated group (Pb/A.Ab group) showed a significant (*P* < 0.05) increase in the levels of Na^+^-K^+^-ATPase, Ca^++^-ATPase and Mg^++^-ATPase in liver and kidney as compared to all Pb and Pb/water group [Tables 5 and 6]. These results indicate a protective and a beneficial effect of A.Ab on membrane bound ATPase activity by virtue of its antioxidant property. Treatment with A.Ab in Pb/A.Ab group showed a significant (*P* < 0.05) decrease in the levels of cholesterol, triglycerides, free fatty acids, and phospholipids in the liver and kidney as compared to Pb and Pb/water groups [Tables 3 and 4]. These results show that, A.Ab has a protective effect on the lead induced changes in the lipid levels.

**Table 3:** Effect of Wormwood extract on levels of total cholesterol, free fatty acids, triglycerides, and phospholipids in liver (mg/100 g tissue)

	At 11 weeks		At 4 weeks		
		
	Untreated	Pb	Untreated/A.Ab	Pb/water	Pb/A.Ab
Total cholesterol	344.39 ± 12.16	576.42 ± 13.85*	345.38 ± 15.91^‡^	546.16 ± 50.70*	373.23 ± 22.30†
Triglycerides	343.5 ± 10.20	613.02 ± 16.36*	350.00 ± 6.86†	584.5 ± 12.66*,†	417.30 ± 28.00*,†
Phospholipids	1508.60 ± 33.56	2976.41 ± 42.28*	1497.29 ± 64.20†	2793.1 ± 27.82*	1633.90 ± 47.83*,†
Free fatty acid	622.5 ± 12.96	924.01 ± 15.46*	613.30 ± 24.71†	802.4 ± 20.71*,†	768.30 ± 58.15*,†

Values are expressed as mean ± SEM (*n* = 6).

* *P* < 0.05, Pb group, untreated/A.Ab group, Pb/water group, and Pb/A.Ab group vs untreated group (control).

† *P* < 0.05, untreated/A.Ab group, Pb/water group, and Pb/A.Ab group are compared vs. Pb group (Student's ‘*t*’ test)

**Table 4:** Effect of wormwood extract on levels of total cholesterol, free fatty acids, triglycerides, and phospholipids in kidney (mg/100 g wet tissue)

	At 11 weeks		At 4 weeks		
		
	Untreated	Pb	Untreated/A.Ab	Pb/water	Pb/A.Ab
Total cholesterol	368.4 ± 12.43	526.1 ± 25.90*	378.2 ± 12.43†	574.2 ± 28.13*	450.3 ± 26.16*,†
Triglycerides	293.5 ± 10.20	503.2 ± 16.36*	300.0 ± 11.86†	498.2 ± 17.91*	424.3 ± 19.25*,†
Phospholipids	1618.6 ± 35.66	2696.4 ± 42.80*	1407.2 ± 44.2*,†	2989.7 ± 24.07*	1833.0 ± 26.82*,†
Free fatty acid	424.0 ± 12.96	738.3 ± 45.46*	433.3 ± 18.22†	812.5 ± 38.21*	587.4 ± 25.46*,†

Values are expressed as mean ± SEM (*n* = 6).

* *P* < 0.05, Pb group, untreated/A.Ab group, Pb/water group, and Pb/A.Ab group vs. untreated group (control).

† *P* < 0.05, untreated/A.Ab group, Pb/water group, and Pb/A.Ab group are compared vs. Pb group (Student's ‘*t*’-test)

**Table 5:** Effect of Wormwood extract on the levels of membrane bound enzymes in liver of rat intoxicated by lead acetate (unit expressed as mM of Pi liberated/mg proteins)

	At 11 weeks		At 4 weeks		
		
	Untreated	Pb	Untreated/A.Ab	Pb/water	Pb/A.Ab
Na^+^-K^+^ ATPase	2.597 ± 0.097	0.772 ± 0.042*	2.723 ± 0.057†	0.653 ± 0.032*,†	1.727 ± 0.133*,†
Ca ^++^-ATPase	1.597 ± 0.070	0.649 ± 0.071*	1.703 ± 0.049*,†	0.526 ± 0.026*,†	1.070 + 0.045*,†
Mg ^++^-ATPase	1.780 ± 0.104	0.947 ± 0.093*	1.642 ± 0.045†	0.855 ± 0.060*	1.197 ± 0.079*,†

Values are expressed as mean ± SEM (*n* = 6).

* *P* < 0.05, Pb group, untreated/A.Ab group, Pb/water group, and Pb/A.Ab group vs. untreated group (control),

† *P* < 0.05, untreated/A.Ab group, Pb/water group, and Pb/A.Ab group are compared vs. Pb group (Student's ‘*t*’ test)

**Table 6:** Effect of Wormwood extract on the levels of membrane bound enzymes in kidney of rat intoxicated by lead acetate (Unit expressed as mM of Pi liberated/mg proteins)

	At 11 weeks		At 4 weeks		
		
	Untreated	Pb	Untreated/A.Ab	Pb/water	Pb/A.Ab
Na ^+^-K^+^ ATPase	3.605 ± 0.078	1.689 ± 0.070*	3.821 ± 0.137†	1.507 ± 0.124*,†	2.444 ± 0.088*,†
Ca ^++^-ATPase	1.942 ± 0.057	0.847 ± 0.023*	1.998 ± 0.031†	0.671 ± 0.042*,†	1.159 ± 0.017*,†
Mg^++^-ATPase	2.837 ± 0.055	1.054 ± 0.049*	3.005 ± 0.062*,†	0.875 ± 0.028*,†	2.341 ± 0.030*,†

Values are expressed as mean ± SEM (*n* = 6).

* *P* < 0.05, Pb group, untreated/A.Ab group, Pb/water group, and Pb/A.Ab group vs. untreated group (control),

† *P* < 0.05, untreated/A.Ab group, Pb/water group, and Pb/A.Ab group are compared vs. Pb group (Student's ‘*t*’-test)

## Discussion

Current views of membrane structure suggest their essential feature to be a bilayer containing a polar shell consisting primarily of phospholipids and free cholesterol. This lipid environment significantly influences the activity of several membrane enzymes.^29^ The molecular mechanisms by which the lipid composition of the membrane bilayer influences enzymes probably involves protein conformational changes due to the physical state of the immediate or boundary lipids and/or restrictions of molecular motion necessary for formation of the transition state. Lipid viscosity is apparently the rate determining factor for the membrane-bound enzymes, polyisoprenoid alcohol phosphokinase^30^ and cytochrome b5.^31^

Our results also indicated that lipid peroxidation and carbonyl were increased in liver and kidney after exposure to lead [Fig. 1]. From the results discussed, it is concluded that lead induces oxidative stress, mainly by disturbing the antioxidant defence of the body. In these cases, the administration of exogenous antioxidants to counteract the proportionate magnitude of the cell injury plays an important role in the treatment of free radical mediated injury or disease.^32^,^33^ Many *in vitro* and *in vivo* studies have shown that MDA increases with lead treatment.^34^ Lead affects the structures of membrane lipids and alters cellular functions like detoxification processes, removal of hydroxyperoxides, protection against effect of ionizing radiations, cellular growth. It is further reported that lead modulates many enzymes and disulfhydril status of proteins.^35^ Lipids are important constituents of the organs. Inorganic lead is a prooxidant and peroxidative damage to cellular membrane lipids and fatty acids leads to membrane fragility and permeability is a likely consequence of lead poisoning.^36^ The increased concentration of cholesterol could result in a relative molecular ordering of the residual phospholipids resulting in a decrease in membrane fluidity.^37^ The increased concentration of free fatty acids in liver and kidney may be due to lipid breakdown and this may cause increased generation of nicotinamide adenine dinucleotide phosphate (NADPH), which results in the activation of NADPH dependent microsomal lipid peroxidation. Liver and kidney phospholipids were increased in Pb group. Phospholipid is present in cell membrane and makes up vast majority of the surface lipoprotein forming a lipid bilayer that acts as an interface with both polar plasma environment and nonpolar lipoprotein of lipoprotein core.^38^ Phospholipids are vital part of biomembrane rich in polyunsaturated fatty acids, which are susceptible substrate for free radicals such as O_2_^·-^ and OH^·^ radicals.^39^ Moreover, the intoxication by lead increased phospholipids levels in tissues and in the levels of triglycerides, indicating the breakdown of fatty acids.^4^

Any alteration in membrane lipid leads to change in membrane fluidity, which in turn alters the ATPase (ATP phosphohydrolase, EC 3.6.1.3) activities and cellular functions. A certain degree of membrane fluidity seems to be essential for Na^+^-K^+^-ATPase. The fluidity of the membrane, to a large extent, is determined by the fatty acids.^40^ Increased enzyme activity was reported with changes in the levels of membrane cholesterol and phospholipids.^37^

In present study levels of Na^+^-K^+^ATPase, Ca^++^-ATPase, and Mg^++^-ATPase were reduced in liver and kidney of lead treated animals (Pb group). This may be because of the changes in the levels of cholesterol, phospholipids, free fatty acids, and triglycerides. The decrease in the levels Na^+^-K^+^-ATPase, Ca^++^-ATPase, and Mg^++^-ATPase could be due to enhanced lipid peroxidation by free radicals in lead-treated animals. Since these membranes bound enzymes are ‘SH’ group containing enzymes, which are lipid dependent.^41^ In the liver and kidney of *Artemisia absinthium* L. extract treated animals (Pb/A.Ab group), the levels of Na^+^-K^+^-ATPase, Ca^++^-ATPase, and Mg^++^-ATPase were restored to near normal. The restored activities of ATPases suggest the ability of wormwood extract to protect the sulfhydryl group from oxidative damage through inhibition of peroxidation of membrane lipids in liver and kidney of rats.

In present study, chronic exposure to lead resulted in decrease in the levels of ATPase and increase in the levels of lipid peroxidation, lipids composition. This may be due to the release of free radicals by lead. Treatment with *Artemisia absinthium* L. extract in rats exposed to lead resulted in the reversal of the levels of ATPase and lipid in live rand kidney of rats.

## Conclusion

In conclusion, lead-induced oxidative damage in liver and kidney and affect membrane function by enhancing lipid peroxidation and carbonyl concentrations, as well as the alteration of ATPase enzymes activity and lipid concentration. Moreover, *Artemisia absinthium* L. had beneficial effect on lipids concentrations and ATPase activities in liver and kidney of rats exposed to lead acetate
